# Do scars caused by past history of *Leishmania major* infection may harbor persistent parasites?

**Published:** 2011-01-10

**Authors:** Rabiaa Manel Sghaier, Fouad Benhnini, Amor Zaatour, Hanène Attia, Ghada Mkannez, Aymen Bali, Fatma Zahra Guerfali, Afif Ben-Salah, Dhafer Laouini, Koussay Dellagi

**Affiliations:** 1 Laboratory of Immuno-Pathology, Vaccinology and Molecular Genetics, Institut Pasteur de Tunis, 1002 Tunis-Belvedere, Tunisia; 2 Laboratory of Medical Epidemiology, Institut Pasteur de Tunis, 1002 Tunis-Belvedere, Tunisia; 3 WHO Collaborating Center for Research and Training in Leishmaniasis, Institut Pasteur de Tunis, 1002 Tunis-Belvedere, Tunisia

## 

Based on the knowledge that a cured infection protects the individual from re-infection, the development of a vaccine to prevent leishmaniasis has been a goal for nearly a century. Indeed, it is generally believed that after healing of leishmaniasis, sterile cure is never achieved and that few residual living parasites will remain sequestered within some host cells that offer them a safe shelter and hence maintain anti-parasite immune memory. This statement is mainly supported by data from experimental leishmaniasis in mice of susceptible or resistant phenotype, in which, live parasites could be recovered from lesions even after healing, and in which disease reactivation can be obtained by immune manipulation even after apparent complete cure.

Whether maintenance of a long-term immune effector memory in humans will also require persistence of live parasites is presently unknown but is very important in the perspective of a vaccine development. Our aim herein was to address the issue of *Leishmania major* parasite persistence *vs.* sterile healing in zoonotic cutaneous leishmaniasis (ZCL) by analyzing biopsies of scars from healed volunteers.

Skin-punch scars’ biopsies (n=59, range of scar age: 1–5 years) have been obtained from volunteers (18–55 years old) living in two ZCL endemic foci, who had a confirmed past history, are clinically cured of ZCL and who gave their written consent. The whole protocol was approved by the local IRB. The specimens were taken under sterile conditions and local anaesthesia. Each specimen was divided into three parts: (*i*) the first sample was processed for quantitative real time PCR, (*ii*) the second was cultured in vitro in enriched medium and (*iii*) the third was inoculated into the footpad of susceptible BALB/c mice which were kept under observation for five months.

For *in vitro* isolation and after microscopic observation for at least 8 weeks, all cultures were found negative. For *in vivo* isolation, biopsy-inoculated mice were killed five months later, and skin fragments, draining lymph nodes and spleen were inoculated into culture medium, observed carefully for at least 8 weeks before being also designated as negative. PCR results were found negative for the majority of biopsies. These results indicate that any potential persistent living parasites after cure are unlikely to be sequestered in ZCL scars. This issue is of importance in strategies for control of leishmaniasis and requires further discussion.

